# Multistage carcinogenesis

**DOI:** 10.1038/sj.bjc.6602318

**Published:** 2004-12-21

**Authors:** R A Weiss

Fifty years ago, Richard Doll and A Bradford Hill reported findings on a cohort of British doctors which demonstrated the strong link between cigarette smoking and lung cancer (Doll and Hill, 1954). A summary of the 50-year follow-up of general mortality in that cohort led by Sir Richard Doll and Sir Richard Peto was recently published (Doll *et al*, 2004) and further analyses specific to cancer mortality will be published in the BJC early in 2005 (Doll *et al*, 2005) with a separate editorial by Peter Boyle. What is less frequently appreciated is that an equally prescient paper by Peter Armitage and Richard Doll was also published 50 years ago in the BJC on multistage carcinogenesis (Armitage and Doll, 1954). Of many papers in the BJC that established new insights into the nature and treatment of cancer, I consider Armitage and Doll (1954) to be one of the great classics, and we are therefore reprinting it in this issue.

Armitage and Doll showed that, for a variety of nonendocrine carcinomas, the incidence of tumours increased with the sixth power of age. From this observation they postulated the multistage theory of carcinogenesis and inferred six to seven independent, sequential and stable events occurring in the cancer lineage before malignancy became manifest. Although Nordling (1953) had recently suggested that such ‘hits’ or events were likely to be successive mutations, Sir Richard tells me that he and Armitage were cautious not to invoke mutation specifically because of the acceptance of the ‘two-stage carcinogenesis’ theory based on experimental tumour production induction using initiating, mutagenic carcinogens followed by inflammatory, nonmutagenic promotors such as phorbol esters. The initiator–promotor model was developed by Isaac Berenblum and Philip Shubik in another early and seminal paper in the BJC (Berenblum and Shubik, 1949). The multistage theory of carcinogenesis has been further developed and refined by Armitage and Doll (1957) themselves, Nick Day (Day and Brown, 1980), Stenback *et al* (1981) and by Suresh Moolgavkar (Moolgavkar, 1978; Luebeck and Moolgavkar, 2002).

The concept of genetic alterations in cancer cells stemmed from Theodor Boveri's (1914) observations on aneuploidy in cancer 90 years ago. Yet at the time of Armitage and Doll's study, despite the evidence that most carcinogens were mutagens, cancer was still not universally accepted as a somatic genetic disease. Sir David Smithers (1962) considered that ‘Cancer is no more a disease of cells than a traffic jam is a disease of cars. A lifetime's study of the internal combustion engine will not explain it’. But in the 50 years since the Armitage and Doll published their theory, the clonal evolution of cancer has been repeatedly demonstrated (Nowell, 1976; Greaves, 2002), and many of the specific genes and signalling pathways have been elucidated that permit tumour cells to proliferate (Fearon and Vogelstein, 1991; Vogelstein and Kinzler, 2004) and spread (Hunter, 2004). These mutations include progressive instability of the cancer cell genome (Loeb, 1991; Cairns, 2002; Feldser *et al*, 2003), which may itself accelerate the number of hits in relation to age. Recent mathematical treatments of somatic mutation in multistage carcinogenesis have emphasized a Darwinian evolutionary perspective to the process (Greaves, 2002; Gatenby and Vincent, 2003; Michor *et al*, 2004). Thus, it seems truly remarkable that the multistage theory of carcinogenesis was established by epidemiologists on the basis of an analysis of the age-specific incidence of common cancers, prior to the development of molecular biology. BJC salutes Sir Richard Doll and Prof Armitage who have continued to contribute actively to our understanding of cancer for 50 years following their pioneering early papers.

The development of familial cancers at relatively young ages can be attributed to the first mutational event being inherited, either as a dominant trait, or as a recessive tumour suppressor gene as elucidated for the *Rb* retinoblastosis locus by Al Knudson (2002). The affected patients are heterozygous for wild-type and mutant *Rb*, which is recessive at the cellular level, but presents as a dominant inheritance in the multicellular human body owing to the high probability that one of the target cells will incur a second, somatic mutation that causes the knockout genotype and hence allows that cell to progress towards the malignant phenotype. One can also interpret the vastly greater cancer rates in the large bowel compared to the small bowel (which has as high a cellular turnover and renewal) as reflecting lower mutation rates in the sterile environment of the small intestine, whereas the colon is a substantial incubator of mutagen-releasing bacteria (Venitt *et al*, 1986). Thus, mutations in multiple genes that alter coding regions or affect expression, account for the many steps in carcinogenesis (Vogelstein and Kinzler, 2004). The functions of the genes concerned involve inhibition of cell proliferation (tumour suppressor genes), positive signalling of proliferation and migration (oncogenes), control of apoptosis, and DNA instability and repair.

Now that the specific mutations and pathways are becoming unravelled (Weinstein *et al*, 1997; Vogelstein and Kinzler, 2004), novel types of therapeutic agents are being avidly sought and pursued. Gleevec (imatinib) acts on the oncoprotein created by a specific chromosome re-arrangement in chronic myelocytic leukaemia (Ross and Hughes, 2004), something that would surely delight Boveri were he alive today. Inhibitors of tyrosine kinase receptors and their downstream signalling pathways present promising targets for translating our scientific understanding of cancer into strategies for prevention and treatment (Ranson, 2004; Ross and Hughes, 2004). Translational and clinical research of this kind is specially welcomed by BJC, as well as investigations in molecular diagnostics, cancer genetics and epidemiology.

One of my own research interests is the development of malignancy in immunocompromised individuals, such as immunosuppressed transplant patients and those with AIDS. Ever since Paul Ehrlich first enunciated the immune surveillance hypothesis of cancer in 1909, it has remained popular among oncologists and adherents of alternative medicine alike, and it is the basis for immunotherapy (Blattman and Greenberg, 2004; Maher and Davies, 2004). However, there is scant evidence for specific T-cell immunity to naturally occurring nonviral cancer in humans, although macrophages may play a role in innate immune surveillance (Alexander, 1976). Analysis of cancer incidence rates in immunocompromised populations reveals greatly increased cancer rates only for those tumours that express nonself-antigens such as those caused by oncogenic viruses: for example, non-Hodgkin's B-cell lymphoma, Kaposi's sarcoma and cervical carcinoma (Boshoff and Weiss, 2002). I suspect that Armitage and Doll were correct all along and that, even in immunosuppressed conditions, potential cancer cells need to clock up six to seven sequential mutations before presenting as cancer. If that is the case, an increase in cancer among immunocompromised patients may be worthy of further investigation, particularly in the older age groups.

This is my final editorial as Editor-in-Chief of the BJC. It has been a privilege and a pleasure to occupy this position for 6 years and I am delighted to welcome my successor, Adrian Harris. I am most grateful to each of the Subject Editors who has served the BJC during this time, as well as the Reviews Editor who has commissioned a lively new series of topical mini-reviews several of which are cited in this editorial. My personal thanks must also go to BJC's business manager, Julia Maidment, whose name does not appear on BJC's title page but who has helped me to guide the journal throughout my period as Editor, and who has ensured the smooth coordination with our publisher, Nature Publishing Group, and our owner, Cancer Research UK. I wish the BJC and advances in the control of cancer every success in the years to come.
